# Efficacy and safety of TACE combined with TKIs and PD-1 inhibitors in HCC patients with prior TIPS

**DOI:** 10.3389/fonc.2025.1570029

**Published:** 2025-04-24

**Authors:** Kai Xiong, Kuiyuan Huang, Yulong Liu, Huajin Pang, Peng Chen, Yalu Zheng, Tengzheng Li, Zhangyun Li, Moran Zhang, Dandan Zheng, Xiaohong Huang, Mingrong Cao, Qiang Li, Junjie Liang, Huizhen Fan, Deju Li, Jian Sun, Zhili Wen, Yuchuan Jiang

**Affiliations:** ^1^ Department of Gastroenterology, The Second Affiliated Hospital of Nanchang University, Nanchang University, Nanchang, Jiangxi, China; ^2^ Department of Vascular Intervention, The First Affiliated Hospital of Jinan University, Jinan University, Guangzhou, Guangdong, China; ^3^ Division of Vascular and Interventional Radiology, Department of General Surgery, Nanfang Hospital, Southern Medical University, Guangzhou, Guangdong, China; ^4^ State Key Laboratory of Organ Failure Research, Guangdong Provincial Key Laboratory of Viral Hepatitis Research, Department of Infectious Diseases, Nanfang Hospital, Southern Medical University, Guangzhou, Guangdong, China; ^5^ Department of Radiation Oncology, The First Affiliated Hospital of Zhejiang University, Hangzhou, Zhejiang, China; ^6^ Department of Pathology, The Second Affiliated Hospital of Nanchang University, Nanchang University, Nanchang, Jiangxi, China; ^7^ Department of Hepatobiliary and pancreatic Surgery, The First Affiliated Hospital of Jinan University, Jinan University, Guangzhou, Guangdong, China; ^8^ Department of Gastroenterology, The People′s Hospital of Yichun City, Yichun, Jiangxi, China; ^9^ Department of Vascular Surgery, Hainan Affiliated Hospital of Hainan Medical University, Haikou, Hainan, China

**Keywords:** hepatocellular carcinoma, tyrosine kinase inhibitors, PD-1 inhibitors, transarterial chemoembolization, transjugular intrahepatic portosystemic shunt

## Abstract

**Purpose:**

To compare the efficacy and safety of TACE combined with TKIs and PD-1 inhibitors between HCC patients with and without prior TIPS

**Methods:**

This retrospective propensity score matching (PSM) study included advanced HCC patients treated with prior TIPS followed by TKIs, PD-1 inhibitors, and TACE between January 2021 and January 2023. Patients were matched with a control group of HCC patients who had not undergone TIPS (non-TIPS). Outcome measures included objective response rate (ORR) using modified RECIST (mRECIST v1.1), disease control rate (DCR), progression-free survival (PFS), overall survival (OS), and safety assessed by CTCAE v5.0.

**Results:**

A total of 172 patients were included before PSM. After PSM, 42 patients with prior TIPS were matched with 71 non-TIPS patients. ORR was 31.0% in the TIPS group and 57.7% in the non-TIPS group (p = 0.007), Both PFS and OS were longer in the non-TIPS group, with a median PFS of 7.9 months for TIPS patients versus 12.3 months for non-TIPS patients (hazard ratio [HR] = 2.253, p < 0.001), and a median OS of 13.5 months versus 21.1 months, respectively (HR = 2.282, p = 0.002). Treatment-related adverse events showed no significant differences between the two groups.

**Conclusion:**

TACE combined with TKIs and PD-1 inhibitors showed lower efficacy in HCC patients with prior TIPS, but it remains a viable option, providing a favorable safety profile and effective disease control.

## Introduction

Hepatocellular carcinoma (HCC), the most prevalent form of primary liver cancer, is the third leading cause of cancer-related mortality worldwide (1). Currently, liver transplantation, surgical resection, and ablation are the primary treatment options for early-stage HCC (2). Due to its insidious onset and the absence of pronounced symptoms in the early stages, the majority of diagnosed HCC patients present with advanced disease. A commonly used therapeutic approach for patients with advanced HCC is regional or systemic chemotherapy (3–5). However, treatment outcomes vary significantly among different subgroups of patients with advanced HCC.

Transjugular intrahepatic portosystemic shunt (TIPS) placement is a widely utilized therapeutic intervention for managing complications associated with portal hypertension, such as variceal hemorrhage and refractory ascites (6). Certain patients with portal hypertension concomitantly have HCC, while others may receive a diagnosis of HCC after TIPS procedures. Moreover, some studies have found that patients with cirrhosis who have undergone TIPS may have increased risk of HCC (7, 8). It is not uncommon for some patients to have undergone TIPS prior to the initiation of anti-HCC therapy. TIPS can modify liver hemodynamics, affect the progression of liver diseases and potentially diminish the efficacy of HCC treatments, thereby complicating the selection of appropriate therapeutic interventions. Thus, it is critical to delineate specific treatment regimens and assess their efficacy for this particular patient cohort.

In recent years, significant advances have been made in the treatment of advanced HCC. Beyond traditional systemic treatments like sorafenib and regorafenib, immunotherapy, particularly anti-PD-1 monotherapy, has revolutionized HCC management, demonstrating improved overall survival and a better safety profile (9). Transarterial chemoembolization (TACE), a key component of locoregional treatments for HCC, and remains essential for patients with manageable tumor burdens and preserved liver function (10). Notably, the integration of systemic treatments with locoregional therapies, such as the combination of TACE with tyrosine kinase inhibitors (TKIs) and anti-PD-1 antibodies, has been shown to have acceptable toxicity profiles and promising anti-tumor efficacy in patients with advanced HCC (11–14). This multi-modal approach, balancing systemic and local treatments, has emerged as a pivotal strategy in the management of advanced HCC, motivating the focus of our study on this therapeutic regimen.

Despite reports demonstrating the safety and efficacy of TACE in HCC patients with prior TIPS (15–17), there is a paucity of research evaluating the combined application of systemic and local therapy in this patient population. The purpose of this study is to evaluate the safety and efficacy profile of TACE combined with TKIs and PD-1 inhibitors in HCC patients with prior TIPS. By comparing outcomes between patients who have undergone TIPS and those who have not (non-TIPS), this study provides important insights into the therapeutic benefits and risks of this combined treatment strategy for HCC patients with prior TIPS.

## Methods

### Study design and patients

**Figure 1:**
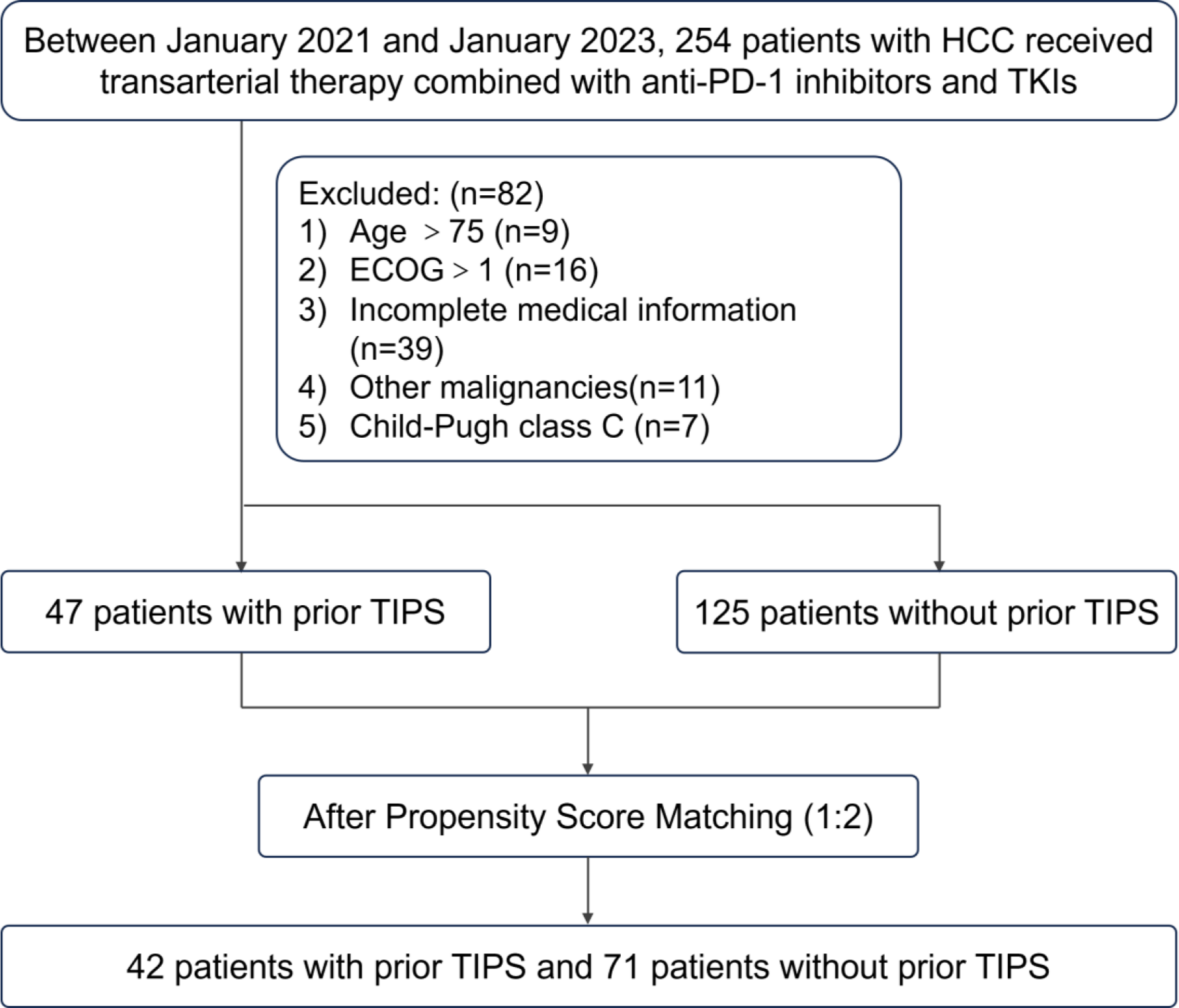
Flow diagram summarizing the patient enrollment process of this study. HCC, hepatocellular carcinoma; TIPS, transjugular intrahepatic portosystemic shunt; TKIs, tyrosine kinase inhibitors; ECOG, eastern cooperative oncology group.

This retrospective study encompassed consecutive HCC patients who received their first treatment as a combination of TACE with tyrosine kinase inhibitors (TKIs) and PD-1 inhibitors at the Second Affiliated Hospital of Nanchang University and the First Affiliated Hospital of Jinan University from January 2021 to January 2023. A total of 172 patients satisfied the eligibility criteria for inclusion (**Figure 1**). All patients included in the study were free from any prior systemic treatment before receiving the combination therapy.

The inclusion criteria specified patients diagnosed with HCC either pathologically or according to the American Association for the Study of Liver Diseases (AASLD) practice guidelines, who were aged 18 years or older and had received the specified combination therapy as their initial treatment. The exclusion criteria included patients aged 75 years or older, those with an Eastern Cooperative Oncology Group (ECOG) performance status greater than 1, Child-Pugh class C liver function, incomplete medical records, or the presence of another malignant tumor.

We enrolled HCC patients with a history of TIPS treatment, divided into two distinct scenarios (1): patients who were initially diagnosed with HCC and symptomatic portal hypertension secondary to cirrhosis, underwent TIPS treatment first, and subsequently received combination therapy with TACE, TKIs, and PD-1 inhibitors after improvements in their ECOG performance status, Child-Pugh classification, and symptoms of portal hypertension; and (2) patients who developed HCC during follow-up after TIPS placement and were treated with the same combination therapy with TACE, TKIs, and PD-1 inhibitors. In both scenarios, the interval between TIPS treatment and the initiation of antitumor therapy was greater than 1 month (18, 19).

The study received approval from the Ethics Committees of both the Second Affiliated Hospital of Nanchang University and the First Affiliated Hospital of Jinan University, ensuring adherence to the ethical standards set forth in the Declaration of Helsinki.

### Propensity score matching

Owing to the retrospective design of this study, there was an inherent risk of both selection bias and residual confounding—common pitfalls where patient enrollment and data collection were not originally structured to ensure balanced characteristics across groups. To address these limitations and to improve the comparability of baseline variables between TIPS and non-TIPS patients, we employed a propensity score matching (PSM) approach.

Patients who met the inclusion criteria and none of the exclusion criteria were divided into two groups based on whether they had undergone a TIPS prior to initiating anti-tumor treatment. Propensity scores were calculated using logistic regression analysis with the MatchIt package in R, considering the following covariates: age, gender, etiology of liver disease, ECOG performance status, Child-Pugh score, Barcelona Clinic Liver Cancer (BCLC) stage, tumor size, alpha-fetoprotein (AFP) levels, vascular invasion, extrahepatic metastasis, previous treatment, and tumor number. Employing a 1:2 nearest neighbor matching algorithm with a caliper width of 0.2 on the logit scale of the propensity score, and without replacement to ensure unique matches, we successfully matched 42 patients who had undergone TIPS with 71 patients who had not, prior to their treatment. This matching ratio was strategically chosen to optimize the balance between the groups and enhance the statistical power of the study′s comparative analysis.

While PSM helps to balance measured confounders, the possibility of unmeasured or unknown factors influencing outcomes cannot be fully excluded, underscoring a persistent limitation inherent to retrospective analyses.

### Treatment protocol

For the TACE procedure, access was established by puncturing the femoral artery using the Seldinger technique. Under the guidance of digital subtraction angiography, a catheter, along with a coaxial microcatheter, was advanced into the hepatic artery. An emulsion containing lipiodol (5–20 mL), epirubicin (50 mg), and lobaplatin (50 mL) was then injected into the tumor′s feeding arteries, followed by embolization using gelatin sponge particles to obstruct the blood flow. The subsequent TACE cycles were contingent upon the tumor’s response to the treatment. TACE was repeated at intervals of 4–6 weeks if viable tumor tissue remained. The procedure was discontinued if the disease progressed beyond the point of beneficial treatment, toxicity reached intolerable levels, or the patient chose to withdraw from the treatment.

The combination therapy of TKIs and PD-1 inhibitors was initiated within 3–5 days following the initial TACE procedure, guided by postprocedural hepatic function recovery and comprehensive evaluation of the patient’s clinical status. TKIs were administered daily in metronomic oral doses as follows: sorafenib at 400 mg, lenvatinib at 8 mg, regorafenib at 80 mg, and donafenib at 200 mg. PD-1 inhibitors were administered intravenously every three weeks, with camrelizumab, sintilimab, and tislelizumab each given at a fixed dose of 200 mg. Pembrolizumab was administered based on body weight at 2 mg/kg, In cases where grade ≥3 adverse events (classified as serious adverse events, SAEs) occurred, the dosages of TKIs and PD-1 inhibitors were subject to reduction, temporary suspension, or complete cessation, depending on the severity of the reaction. The management of TKIs and PD-1 inhibitors, including any dose adjustments, suspensions, or discontinuations, adhered strictly to local care standards and the stipulations of the approved product labeling.

### Efficacy and safety assessment

Diagnostic, clinical, and radiological details were retrospectively retrieved from patient records. Every six weeks, patients underwent upper abdomen-enhanced CT or MRI, along with chest CT scans. Laboratory tests were conducted every three weeks. All imaging data were independently reviewed by two experienced radiologists. In instances of disagreement, a third, senior radiologist was consulted to make the final adjudication. Tumor response was evaluated with the best overall response using the modified Response Evaluation Criteria in Solid Tumors (mRECIST v1.1), which includes CR, partial response (PR), stable disease (SD), and progressive disease (PD).

The primary endpoints of this study were Overall Survival (OS) and Progression-Free Survival (PFS). OS was defined as the interval from the initiation of therapy to death from any cause or to the last date the patient was known to be alive. PFS was measured from the commencement of treatment to the first occurrence of disease progression or death, whichever occurred first. Secondary endpoints comprised the Objective Response Rate (ORR) and Disease Control Rate (DCR). ORR was defined as the proportion of patients achieving a CR or a PR, with responses required to be sustained for at least four weeks after the initial radiological assessment. DCR included ORR along with the proportion of patients exhibiting SD. Adverse events were monitored and classified according to the National Cancer Institute Common Terminology Criteria for Adverse Events, version 5.0 (NCI-CTCAE v5.0).

### Statistics analysis

Statistical analyses were performed using SPSS (version 26, Chicago, IL, USA). Continuous variables were compared utilizing either the Student′s t-test or the Mann-Whitney U test, while categorical variables were assessed using the chi-square test or Fisher’s exact test. Survival outcomes were estimated using the Kaplan-Meier method and compared via log-rank tests. The efficacy of PSM was verified by assessing standardized mean differences, all of which were maintained at or below 0.1, demonstrating adequate balance between the matched groups. All statistical tests were two-sided, with a significance level set at P < 0.05.

## Results

### Baseline patient characteristics

Before PSM, basal clinicopathologic characteristics of HCC patients with and without a TIPS who underwent TACE combined with TKIs and PD-1 inhibitors are shown in **Table 1**. In the TIPS group, patients had a mean age of 55.3 years, and 43 patients (91.5%) were male. Hepatitis B virus infection was identified in 42 patients (89.4%). An ECOG performance status of 0 was observed in 21 patients (44.7%), and BCLC stage C was present in 34 patients (72.3%). Child-Pugh class A was noted in 29 patients (61.7%). Twenty patients (42.6%) had a tumor size less than 5 cm, and vascular invasion was exhibited by 30 patients (63.8%). The majority of patients (36, 76.6%) had not received previous treatments. In the non-TIPS group, patients had a mean age of 52.2 years, with 109 patients (87.2%) being male. Hepatitis B virus infection was present in 114 patients (91.2%). An ECOG score of 0 was observed in 65 patients (52.0%), and BCLC stage A or B was noted in 48 patients (38.4%). Child-Pugh class A was found in 103 patients (82.4%). Twenty-eight patients (22.4%) had a tumor size less than 5 cm, and vascular invasion was present in 71 patients (56.8%). Most patients (94, 75.2%) had not undergone previous treatment.

**Table 1:** Clinicalpathologic characteristics of HCC cohorts before and after PSM.

Variable	Before PSM			After PSM		
	TIPS (N=47)	Non-TIPS (N=125)	^a^P-value	TIPS (N=42)	Non-TIPS (N=71)	^a^P-value
Age, years Mean (SD)	55.3 (7.95)	52.2 (11.4)	0.056	54.5 (8.10)	54.7 (10.6)	0.988
Gender			0.434			0.846
Female	4 (8.5%)	16 (12.8%)		4 (9.5%)	6 (8.5%)	
Male	43 (91.5%)	109 (87.2%)		38 (90.5%)	65 (91.5%)	
ECOG score			0.392			0.907
0	21 (44.7%)	65 (52.0%)		20 (47.6%)	33 (46.5%)	
1	26 (55.3%)	60 (48.0%)		22 (52.4%)	38 (53.5%)	
BCLC			0.247			0.729
A	5 (10.6%)	11 (8.8%)		5 (11.9%)	7 (9.9%)	
B	8 (17.0%)	37 (29.6%)		8 (19.0%)	18 (25.4%)	
C	34 (72.3%)	77 (61.6%)		29 (69.0%)	46 (64.8%)	
Child-Pugh			0.004			0.563
A	29 (61.7%)	103 (82.4%)		28 (66.7%)	51 (71.8%)	
B	18 (38.3%)	22 (17.6%)		14 (33.3%)	20 (28.2%)	
Hepatitis B infection			0.711			0.234
No	5 (10.6%)	11 (8.8%)		5 (11.9%)	4 (5.6%)	
Yes	42 (89.4%)	114 (91.2%)		37 (88.1%)	67 (94.4%)	
Tumor size			0.009			0.718
<5 cm	20 (42.6%)	28 (22.4%)		15 (35.7%)	23 (32.4%)	
≥5 cm	27 (57.4%)	97 (77.6%)		27 (64.3%)	48 (67.6%)	
Tumor number			0.933			0.819
Single	18 (38.3%)	47 (37.6%)		18 (42.9%)	32 (45.1%)	
Multiple	29 (61.7%)	78 (62.4%)		24 (57.1%)	39 (54.9%)	
Extrahepatic metastasis			0.856			0.878
No	33 (70.2%)	86 (68.8%)		29 (69.0%)	50 (70.4%)	
Yes	14 (29.8%)	39 (31.2%)		13 (31.0%)	21 (29.6%)	
AFP			0.441			0.978
<400 ng/ml	23 (48.9%)	53 (42.4%)		20 (47.6%)	34 (47.9%)	
≥400 ng/ml	24 (51.1%)	72 (57.6%)		22 (52.4%)	37 (52.1%)	
Vascular invasion			0.404			0.913
No	17 (36.2%)	54 (43.2%)		17 (40.5%)	28 (39.4%)	
Yes	30 (63.8%)	71 (56.8%)		25 (59.5%)	43 (60.6%)	
Previous treatment			0.510			0.845
No	36 (76.6%)	94 (75.2%)		33 (78.6%)	52 (73.2%)	
Resection	3 (6.4%)	12 (9.6%)		3 (7.1%)	8 (11.3%)	
Ablation	2 (4.3%)	10 (8.0%)		2 (4.8%)	5 (7.0%)	
Others	6 (12.8%)	9 (7.2%)		4 (9.5%)	6 (8.5%)	

PSM, propensity score matching; TIPS, transjugular intrahepatic portosystemic shunt; ECOG, Eastern Cooperative Oncology Group; BCLC, Barcelona Clinic Liver Cancer; AFP, alpha-fetalprotein.

a Statistical significance was assessed with the Chi-square test.

**Figure 2:**
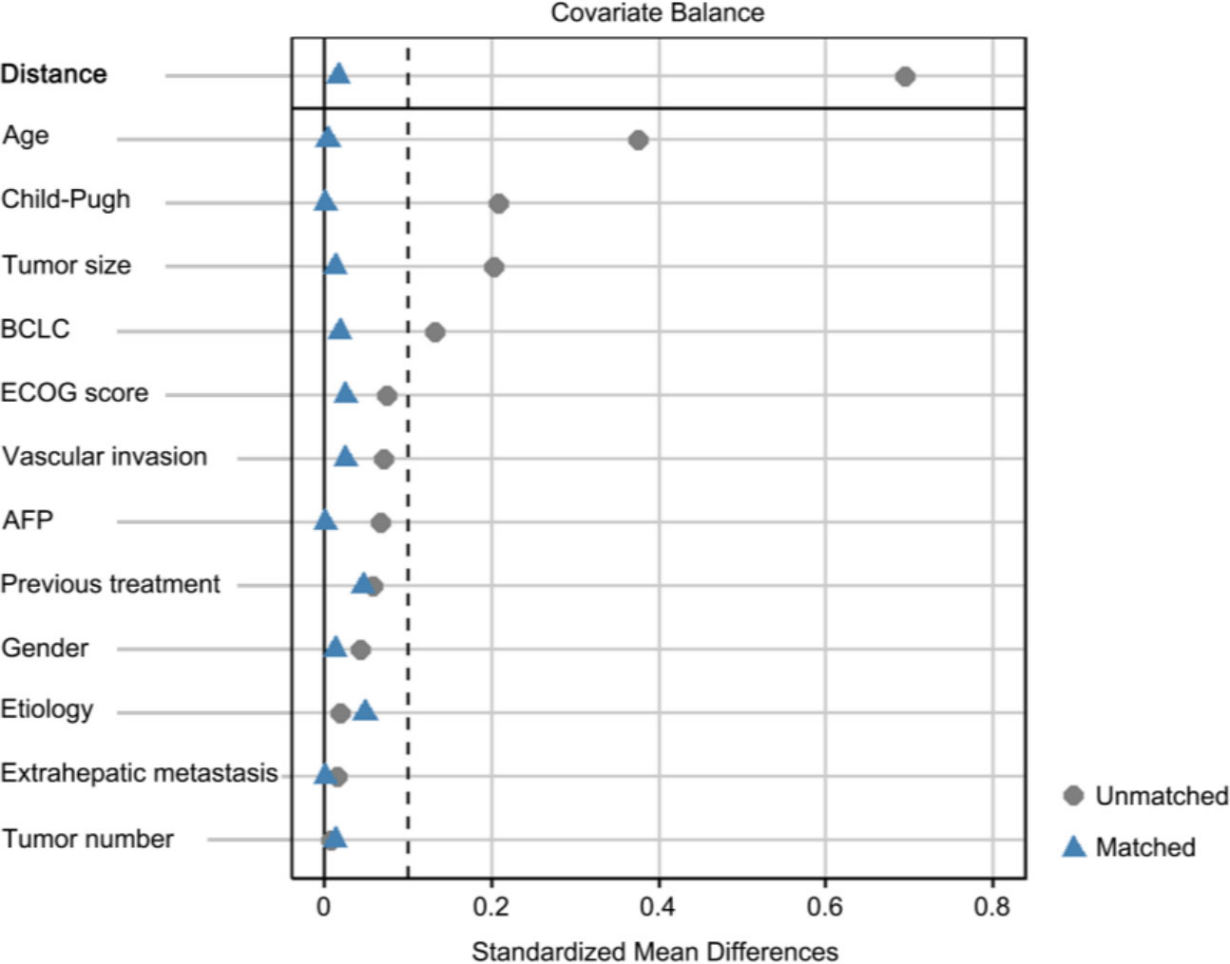
Standardized mean differences (SMDs) of covariates before and after propensity matching. The dashed vertical line represents a SMD of 0.1. BCLC, barcelona clinic liver cancer; AFP, α-fetoprotein.

After PSM, almost all differences before matching disappeared, as indicated in **Table 1** and demonstrated in **Figure 2**, which shows the standardized mean differences (SMDs) of the covariates between the TIPS and non-TIPS groups before and after propensity score matching. The dashed vertical line in **Figure 2** represents an SMD of 0.1, where values beyond this threshold indicate a significant imbalance. Prior to matching, several covariates, including age, Child-Pugh class, tumor size, and BCLC stage had SMDs greater than 0.1, indicating imbalance between the groups. The Distance variable, representing the logit distance between matched pairs based on their propensity scores, was also above 0.1 before matching, highlighting substantial initial differences in propensity scores between the groups. After matching, all covariates, including Distance, were balanced with SMDs below 0.1, indicating successful alignment of the groups in terms of clinical and pathological characteristics. The treatment patterns are displayed in **Table 2**, and no significant differences were observed in the types of TACE cycles, TKIs, or ICIs between the two groups.

**Table 2:** Treatment pattern.

N (%)	TIPS (n=42)	Non-TIPS (n=71)	^a^P-value
Number of TACE			0.604
≤2times	15 (35.7%)	22 (31.0%)	
>2times	27 (64.3%)	49 (69.0%)	
Tyrosine kinase inhibitor			0.483
Lenvatinib	22 (52.4%)	42 (59.2%)	
Others	20 (47.6%)	29 (40.8%)	
Duration of TKIs (months)			0.686
≤3	9 (21.4%)	13 (18.3%)	
>3	33 (78.6%)	58 (81.7%)	
PD-1 inhibitors			0.737
Camrelizumab	17 (40.5%)	24 (33.8%)	
Sintilimab	15 (35.7%)	26 (36.6%)	
Tislelizumab	8 (19.0%)	19 (26.8%)	
Pembrolizumab	2 (4.8%)	2 (2.8%)	
Cycles of PD-1 inhibitors			0.740
≤3	10 (23.8%)	15 (21.1%)	
>3	32 (76.2%)	56 (78.9%)	

TACE, transarterial chemoembolization.

a Statistical significance was assessed with the Chi-square test.

### Tumor response and survival

**Figure 3:**
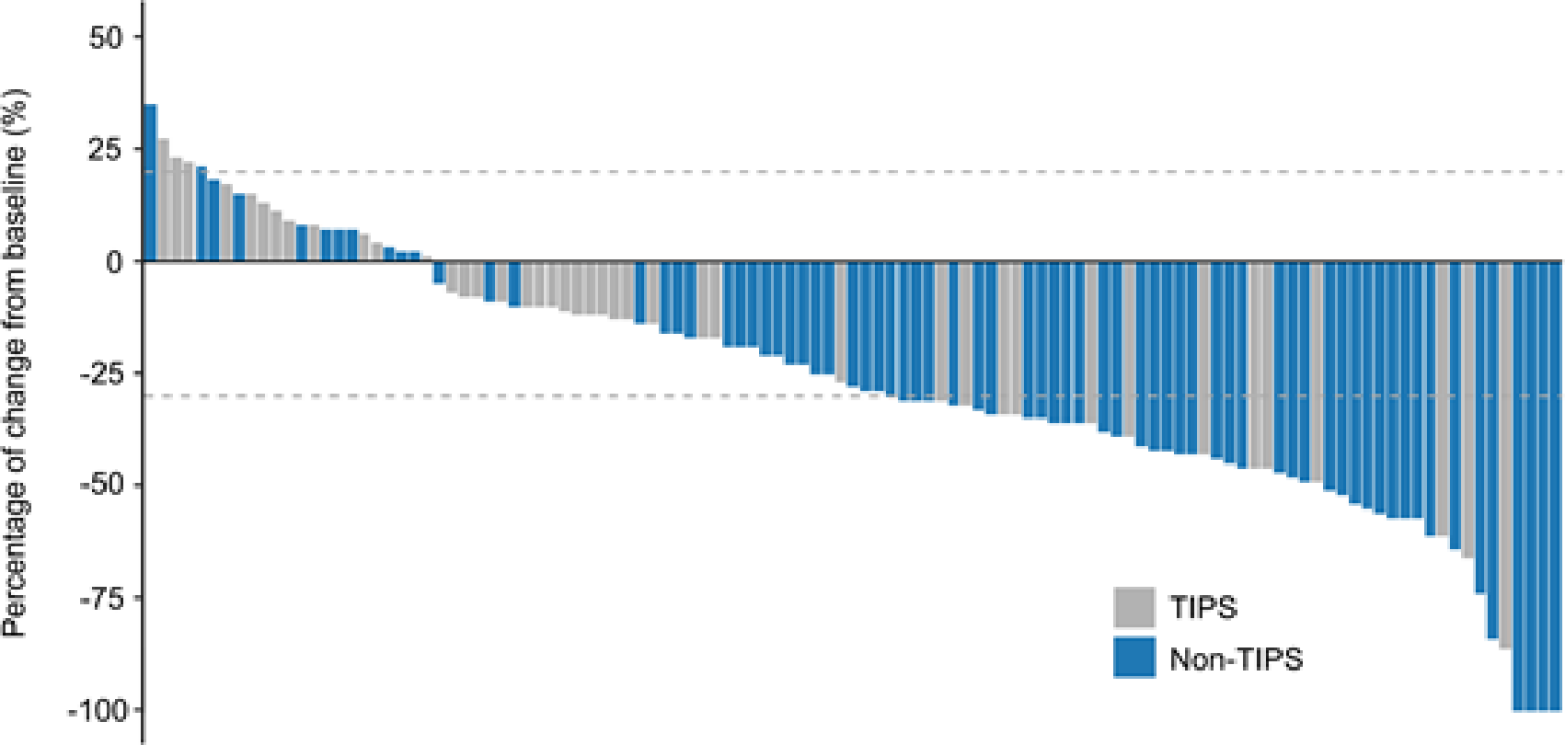
Tumor response evaluation based on the mRECIST in HCC patients with or without TIPS receiving TACE combined with PD-1 inhibitors and TKIs. mRECIST, modified response evaluation criteria in solid tumors.

In the entire cohort, the ORR and DCR were 47.8% and 89.4%, respectively, including 3.5% CR, 44.2% PR, 41.6% SD, and 10.6% PD. There was a significant difference in the overall tumor response distribution between the TIPS and non-TIPS groups (p = 0.031). The non-TIPS group showed a higher proportion of ORR compared to the TIPS group (57.7% vs. 31.0%, p = 0.007). The DCR was 91.5% in the non-TIPS group and 85.7% in the TIPS group, with no significant difference between the two (p = 0.357). The TIPS group had an SD rate of 54.8% and a PD rate of 14.3%, while the non-TIPS group had an SD rate of 33.8% and a PD rate of 8.5% (**Table 3**). Among patients with PD, locoregional progression occurred in 2 non-TIPS and 3 TIPS patients, while new metastatic lesions developed in 4 non-TIPS and 3 TIPS patients. Changes in intrahepatic target lesion size are illustrated in the waterfall plot (**Figure 3**).

**Table 3:** Summary of the best response.

N (%)	TIPS (N=42)	Non-TIPS (N=71)	^a^P-value
Tumor response			
CR	0 (0%)	4 (5.6%)	0.031
PR	13 (31.0%)	37 (52.1%)	
SD	23 (54.8%)	24 (33.8%)	
PD	6 (14.3%)	6 (8.5%)	
ORR	13 (31.0%)	41 (57.7%)	0.007
DCR	36 (85.7%)	65 (91.5%)	0.357

CR, complete response; PR, partial response; SD, stable disease; PD, progressive disease; ORR, objective response rate; DCR, disease control rate.

a Statistical significance was assessed with the Chi-square test.

**Figure 4:**
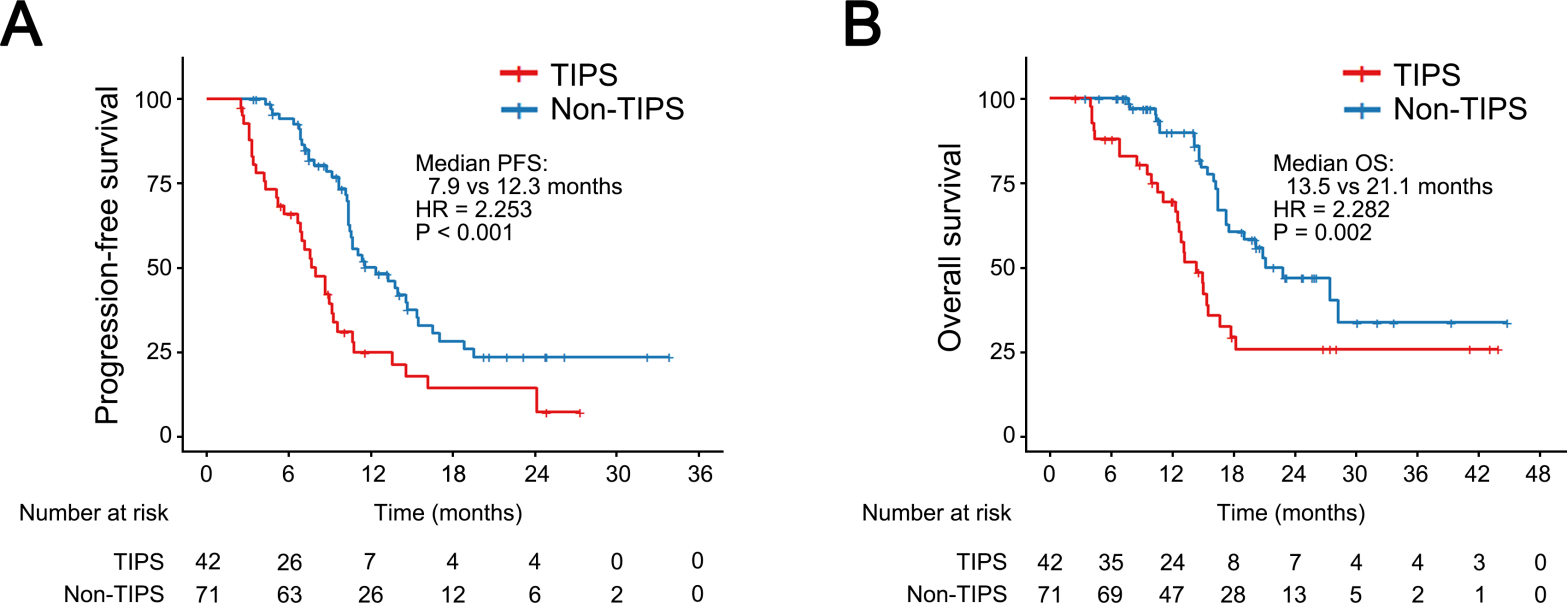
Kaplan–Meier curves for progression-free survival **(A)** and overall survival **(B)** in HCC patients with or without prior TIPS. CI, confidence interval; HR, hazard ratio.

At the time of data cut-off, 42 patients in the non-TIPS group had experienced disease progression, and 27 had died, while in the TIPS group, 34 patients had disease progression and 26 had died. Kaplan-Meier analysis revealed that the non-TIPS group had 1-year and 2-year OS rates of 89.7% and 46.5%, respectively, while the TIPS group had rates of 69.2% and 25.1%. Regarding PFS, the 1-year and 2-year rates were 50.0% and 23.4% in the non-TIPS group and 24.7% and 7.6% in the TIPS group. The median PFS in the TIPS group was 7.9 months, significantly shorter than the 12.3 months observed in the non-TIPS group (HR = 2.253; 95% CI, 1.429–3.552; P < 0.001; **Figure 4A**). Similarly, the non-TIPS group had a median OS of 21.1 months, whereas the TIPS group had 13.5 months (HR = 2.282; 95% CI, 1.327–3.926; P = 0.002; **Figure 4B**).

**Figure 5:**
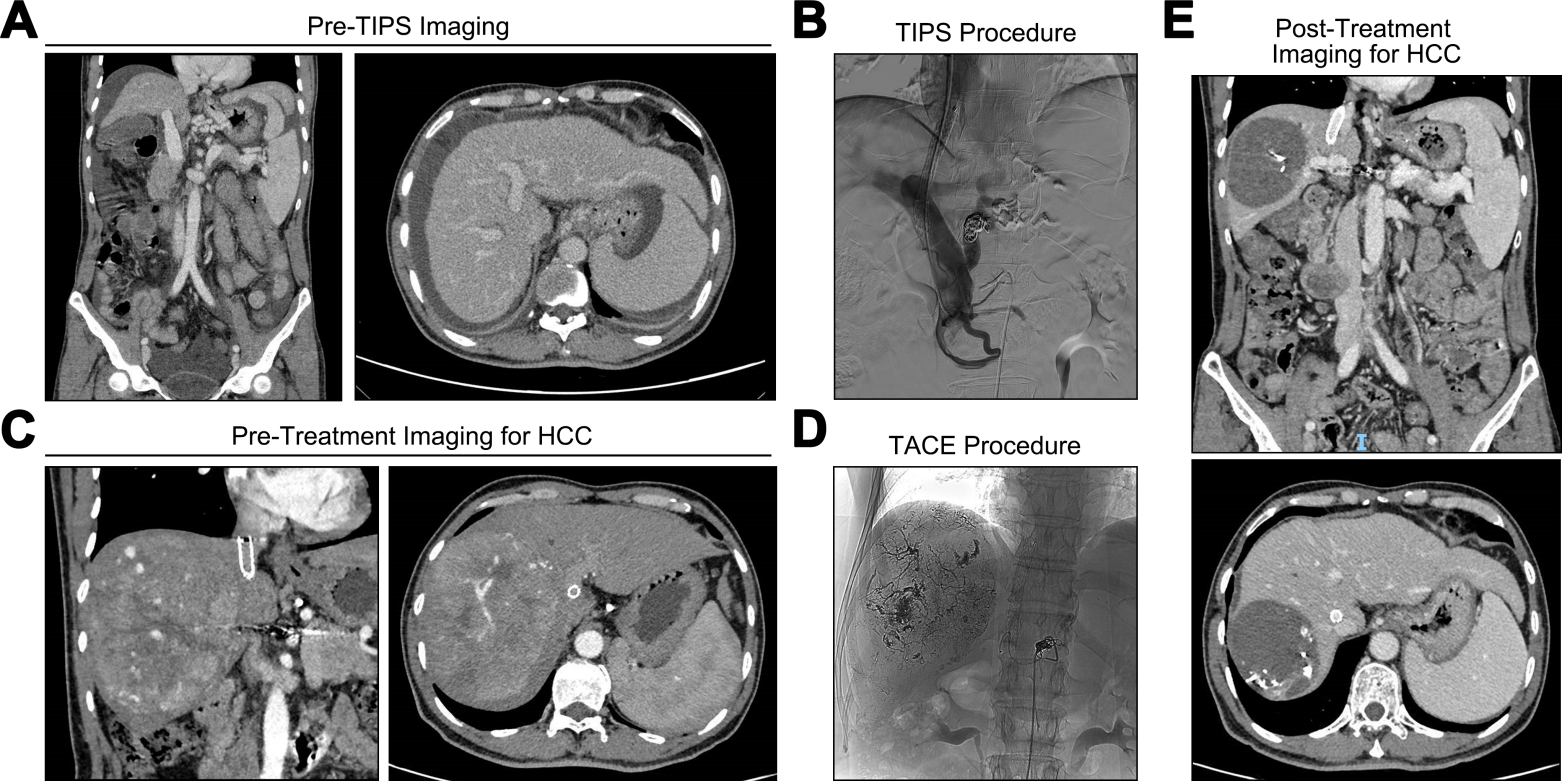
Case of prior TIPS combined with subsequent antitumor treatment. **(A)** refractory ascites and varices before TIPS procedure. **(B)** TIPS was completed. **(C)** abdominal computed tomography (CT) scan suggested a space occupying lesion in the liver. **(D)** the digital subtraction angiography during TACE treatment. **(E)** the abdominal CT findings after the combination of TACE with immunotherapy.

With the extensive use of TIPS in patients complicated with portal hypertension due to its safety and effectiveness, the local-systemic therapy of HCC in patients who have undergone TIPS requires individualized evaluation. Although a higher tumor response rate among patients in the non-TIPS group was observed, the selection of appropriate patients with prior TIPS for anti-HCC therapy could also yield unexpectedly favorable clinical outcomes. As shown in **Figure 5**, a 58-year-old male patient with portal hypertension underwent TIPS procedure for refractory ascites. The liver function improved, as evidenced by a reduction in the child-pugh score from grade B to grade A, and ascites disappeared completely after TIPS. One year later, the patient was diagnosed with HCC and subsequently received a combination of TACE with immunotherapy. Notably, the maximum transverse diameter of the tumor decreased from 13.5 cm to 6.1 cm, with no evidence of intrahepatic or extrahepatic recurrence or metastasis following the combined therapeutic approach. A partial response was sustained throughout the follow-up period according to mRECIST v1.1.

### Safety

There were no treatment-related deaths in both groups. The distributions of treatment-related adverse events (TRAEs) were presented in **Table 4**. The frequency of AEs was comparable between the two treatment groups, with the majority of events being of mild severity. Increased aspartate aminotransferase, Increased alanine aminotransferase, neutropenia, thrombocytopenia, abdominal pain, fatigue and weight loss were the most frequent AEs. The most frequently observed grade 3–4 adverse events (AEs), including Increased liver enzyme levels, neutropenia, fatigue and weight loss were slightly higher in the TIPS group, but the difference was not statistically significant. Interestingly, immune-related myocarditis was observed in 1 patient in the non-TIPS group but not in the TIPS group. Overall, the risk of TRAEs remained unchanged with the implementation of the TIPS procedure prior to anti-HCC treatments.

**Table 4:** Treatment-related adverse events ^a^.

Adverse event	TIPS (N=42)		Non-TIPS (N=71)		^a^P-value P-value	
n%	Any grade	Grade 3–4	Any grade	Grade 3–4	Any grade	Grade 3–4
Neutropenia	22 (52.4%)	4 (9.5%)	33 (46.5%)	6 (8.5%)	0.680	1.000
Anaemia	7 (16.7%)	0 (0%)	9 (12.7%)	2 (2.8%)	0.757	0.529
Thrombocytopenia	19 (45.2%)	5 (11.9%)	35 (49.3%)	11 (15.5%)	0.824	0.803
ALT increased	31 (73.8%)	7 (16.7%)	46 (64.8%)	9 (12.7%)	0.432	0.757
AST increased	24 (57.1%)	10 (23.8%)	47 (66.2%)	12 (16.9%)	0.447	0.515
Fever	18 (42.9%)	0 (0%)	36 (50.7%)	1 (1.4%)	0.540	1.000
Abdominal pain	23 (54.8%)	2 (4.8%)	41 (57.7%)	7 (9.9%)	0.910	0.543
Weight loss	16 (38.1%)	2 (4.8%)	30 (42.3%)	3 (4.2%)	0.813	1.000
Hyper bilirubinacemia	13 (31%)	1 (2.4%)	26 (36.6%)	4 (5.6%)	0.684	0.734
Proteinuria	12 (28.6%)	1 (2.4%)	19 (26.8%)	3 (4.2%)	1.000	1.000
Hypertension	27 (64.3%)	4 (9.5%)	40 (56.3%)	8 (11.3%)	0.527	1.000
Fatigue	29 (69%)	3 (7.1%)	32 (45.1%)	2 (2.8%)	0.023	0.544
Nausea	20 (47.6%)	0 (0%)	27 (38%)	0 (0%)	0.422	1.000
Vomiting	14 (33.3%)	0 (0%)	16 (22.5%)	0 (0%)	0.300	1.000
Diarrhea	8 (19%)	2 (4.8%)	12 (16.9%)	1 (1.4%)	0.973	0.641
Hand-foot skin reaction	11 (26.2%)	1 (2.4%)	20 (28.2%)	2 (2.8%)	0.992	1.000
Rash	9 (21.4%)	0 (0%)	13 (18.3%)	1 (1.4%)	0.874	1.000
Immune-related yocarditis	0 (0%)	0 (0%)	1 (1.4%)	0 (0%)	1.000	1.000

a Statistical significance was assessed with the Chi-square test.

ALT, alanine aminotransferase; AST, aspartate aminotransferase.

b Listed are adverse events, as defined by the National Cancer Institute Common Terminology Criteria (version 5.0).

## Discussion

In this retrospective study, the efficacy and safety of TACE combined with TKIs and PD-1 inhibitors were evaluated in HCC patients with prior TIPS, in comparison to those without prior TIPS. Following the 1:2 PSM analysis, the baseline characteristics between the groups were well-balanced. While the non-TIPS group had a higher ORR and longer median OS and PFS, the therapy of TACE with TKIs and PD-1 inhibitors still showed promise in the TIPS group. Importantly, both cohorts exhibited manageable safety profiles, with comparable incidences of grade 3-4 adverse events (AEs).

Currently, TACE combined with TKIs and PD-1 inhibitors has demonstrated significant efficacy in advanced HCC treatment, achieving notable response rates and survival benefits (20–22). However, few studies have evaluated its effectiveness specifically in HCC patients with prior TIPS. Our findings underscore critical considerations for managing HCC patients with prior TIPS. Although the TIPS group exhibited reduced efficacy compared to non-TIPS patients, the combination of TACE with TKIs and PD-1 inhibitors remains a viable option, particularly given its preserved safety profile and potential for disease control. These results provide valuable insights for optimizing treatment strategies for HCC patients who have previously undergone TIPS, with particular relevance to two specific patient populations (1): patients with pre-existing HCC and symptomatic portal hypertension, in whom TIPS prior to anti-tumor therapy not only alleviates life-threatening complications but may also stabilize liver function, enabling subsequent treatment initiation; and (2) patients with *de novo* HCC detected during TIPS follow-up, for whom this regimen offers therapeutic utility if liver function permits (Child-Pugh A/B), albeit necessitating closer surveillance due to an increased risk of disease progression. Importantly, the dosing and administration intervals for medications in this treatment strategy can be guided by established TACE-TKIs-PD-1 inhibitors combination therapy protocols (21).

The combinative therapy of TACE with TKIs and PD-1 inhibitors provided superior survival benefits for HCC patients who have undergone TIPS compared to those treated with TACE alone, exceeding outcomes reported in previous studies (15, 17). While these findings are promising and require further validation through rigorously designed controlled trials, the presence of indirect evidence provides additional support for these results. The OS and PFS observed in the non-TIPS group undergoing triple therapy closely mirror outcomes published in existing literature, confirming the alignment of our therapeutic strategy with recognized treatment modalities. Furthermore, despite variations in the treatment regimens from those used in previous studies, the non-TIPS group exhibited superior outcomes compared to the TIPS group, which is consistent with trends noted in the broader research literature (15). Collectively, these elements reinforce the validity of the superior results observed in the TIPS group, underscoring the robustness of our treatment protocol and its potential applicability across diverse patient subgroups.

The observed variation in response rates between the non-TIPS and TIPS groups may be partly explained by artery-to-portal vein (arterioportal) shunting. A prior study revealed that approximately 30% of the flow through TIPS could be attributed to direct arterioportal shunting, as quantified by measuring blood flow in both the shunt and the portal vein (23). This phenomenon may compromise the efficacy of TACE by diverting chemoembolic agents away from the target lesion, thereby reducing their local concentration and therapeutic impact. Moreover, arterioportal shunting can exacerbate tumor hypoxia, which promotes immunosuppression and resistance to anti-angiogenic therapies, potentially diminishing the anti-tumor effects of TKIs and anti-PD-(L)1 immunotherapy (24–26). Beyond these local effects, TIPS-induced alterations in hepatic blood flow may also affect the pharmacokinetics of systemically administered drugs (27). For instance, changes in portal venous pressure and shunt flow can reduce intrahepatic drug retention time, altering the metabolism, clearance, and overall bioavailability of TKIs and PD-1 inhibitors. Collectively, these factors—arterioportal shunting, hypoxia, changes in the immune microenvironment, and altered pharmacokinetics—likely contribute to the reduced efficacy of TACE, TKIs, and PD-1 inhibitors in patients with TIPS. Further research is needed to clarify these mechanisms in detail and to explore strategies for optimizing drug delivery and dosing in this patient population.

No treatment-related fatalities were reported in this study. The rate of TRAEs, like abdominal pain, increased liver enzyme levels, haematological toxicity, hypertension, proteinuria, and weight loss were consistent with those reported in previous studies (12, 28). Additionally, there existed no statistically significant difference in TRAES between the TIPS group and the non-TIPS group. Anticoagulant therapy, specifically rivaroxaban, was administered to patients with prior TIPS who had portal vein thrombosis identified before the TIPS procedure, for a duration of 1 to 3 months, and while there is no evidence suggesting that rivaroxaban affects the efficacy of TACE combined with PD-1 inhibitors and TKIs, its use may theoretically increase the risk of bleeding during combined anti-tumor therapy, though no severe bleeding events were observed in our study population, indicating that this risk is manageable. Theoretically, TACE could exacerbate hepatic dysfunction in patients with prior TIPS due to the reduction in portal venous flow caused by TIPS-induced disruption of arterial vasculature (29), but only a tendency toward increased Grade 3-4 liver transaminase elevations was observed in the TIPS group compared to the non-TIPS group (p > 0.05), a difference potentially attributable to the optimal baseline hepatic function of the patients, ultimately demonstrating that the combination of TKIs and PD-1 inhibitors with TACE is safe in HCC patients with prior TIPS.

There are several limitations to our study. Firstly, the retrospective design and lack of randomization may introduce biases, such as selection bias and unmeasured confounding, even though there were no significant differences in baseline characteristics after propensity score matching. Selection bias could arise from the non-random inclusion of patients, as those with more favorable prognoses or better overall health may have been more likely to receive the combination therapy. Additionally, unmeasured confounding factors, such as variations in patient adherence to treatment, differences in tumor biology, or underlying comorbidities, could influence the outcomes but were not accounted for in our analysis. Secondly, the sample size for the TIPS group was relatively limited, whereas the potential cohort of non-TIPS patients was substantially larger. This imbalance in sample size may limit the statistical power of our findings, particularly in subgroup analyses. Hence, multicenter, prospective randomized studies are needed to validate our findings and address these limitations. Lastly, Hepatitis B virus was identified as the principal etiological factor contributing to the development of HCC in this study. Therefore, the generalizability of our results to the broader population of HCC patients requires further investigation.

In conclusion, while the efficacy of TACE combined with TKIs and PD-1 inhibitors appears reduced in HCC patients with prior TIPS compared to those without prior TIPS, the combination therapy still presents a viable treatment option for TIPS patients. This viability is underscored by its favorable safety profile and DCR, making it a suitable therapeutic choice for managing HCC in this specific patient.

## Data Availability

The raw data supporting the conclusions of this article will be made available by the authors, without undue reservation.
